# Genetic and phenotypic evidence of the *Salmonella enterica* serotype Enteritidis human-animal interface in Chile

**DOI:** 10.3389/fmicb.2015.00464

**Published:** 2015-05-15

**Authors:** Patricio Retamal, Marcela Fresno, Catherine Dougnac, Sindy Gutierrez, Vanessa Gornall, Roberto Vidal, Rolando Vernal, Myriam Pujol, Marlen Barreto, Daniel González-Acuña, Pedro Abalos

**Affiliations:** ^1^Departamento de Medicina Preventiva, Facultad de Ciencias Veterinarias y Pecuarias, Universidad de ChileSantiago, Chile; ^2^Emerging and Remerging Zoonosis Research NetworkSantiago, Chile; ^3^Programa de Doctorado en Ciencias Silvoagropecuarias y Veterinarias, Universidad de ChileSantiago, Chile; ^4^Programa de Microbiología, Facultad de Medicina, Universidad de ChileSantiago, Chile; ^5^Departamento de Odontología Conservadora, Facultad de Odontología, Universidad de ChileSantiago, Chile; ^6^Facultad de Ciencias de la Salud, Universidad AutónomaSantiago, Chile; ^7^Facultad de Ciencias Veterinarias, Universidad de ConcepciónChillán, Chile

**Keywords:** *Salmonella enterica*, Enteritidis, humans, poultry, seabirds, Chile

## Abstract

*Salmonella enterica* serotype Enteritidis is a worldwide zoonotic agent that has been recognized as a very important food-borne bacterial pathogen, mainly associated with consumption of poultry products. The aim of this work was to determine genotypic and phenotypic evidence of *S*. Enteritidis transmission among seabirds, poultry and humans in Chile. Genotyping was performed using PCR-based virulotyping, pulse-field gel electrophoresis (PFGE) and multi-locus sequence typing (MLST). Pathogenicity-associated phenotypes were determined with survival to free radicals, acidic pH, starvation, antimicrobial resistance, and survival within human dendritic cells. As result of PCR and PFGE assays, some isolates from the three hosts showed identical genotypic patterns, and through MLST it was determined that all of them belong to sequence type 11. Phenotypic assays show diversity of bacterial responses among isolates. When results were analyzed according to bacterial host, statistical differences were identified in starvation and dendritic cells survival assays. In addition, isolates from seabirds showed the highest rates of resistance to gentamycin, tetracycline, and ampicillin. Overall, the very close genetic and phenotypic traits shown by isolates from humans, poultry, and seabirds suggest the inter-species transmission of *S*. Enteritidis bacteria between hosts, likely through anthropogenic environmental contamination that determines infection of seabirds with bacteria that are potentially pathogenic for other susceptible organism, including humans.

## Introduction

Worldwide, reported human *Salmonella* infections are caused by many serotypes, although at present, the highest incidence is represented by *S. enterica* serotype Enteritidis (Hendriksen et al., 2011; Jackson et al., 2013). The changing epidemiology of *S*. Enteritidis infection over the last 20 years has allowed this serotype to become the most prevalent among *S. enterica* serotypes, and is therefore considered an emergent pathogen. Sources of *S*. Enteritidis are generally associated with commercial poultry products, mainly undercooked eggs and meat (Jackson et al., 2013). In Chile, *S*. Enteritidis emerged in 1994, causing food-borne disease in humans and is now included in surveillance systems for both animal and public health services (Fernandez et al., 2003). However, its current endemic condition has shown increasing incidence during the last years, in spite of sanitary regulations and a previous successful campaign against *S*. Typhi (Fica et al., 2012). The epidemiological link between poultry and human *S*. Enteritidis infection has been confirmed through genotypic analysis that has shown the presence of two major *S*. Enteritidis subtypes distributed between both hosts in the Chilean territory (Fernandez et al., 2003). However, because some clinical strains show unique genetic patterns, it seems that infection of humans is also coming from different unknown sources.

Regarding wildlife hosts, seabirds have been associated elsewhere with zoonotic serotypes of *S. enterica*, with evidence that suggests a direct bacterial transmission either among themselves, with other animals or humans (Reche et al., 2003; Pennycott et al., 2006; Dhama et al., 2008; Skov et al., 2008; Horton et al., 2013; Gruszynski et al., 2014). In addition, along the Chilean coast, zoonotic and multi-drug resistant (MDR) *Salmonella* strains have been detected in seashore animals, specifically seabirds and pinnipeds (Lopez-Martin et al., 2011; Sturm et al., 2011; Rodriguez et al., 2012a; Fresno et al., 2013).

The extended geographical distribution, host range and genome plasticity of *S*. Enteritidis have determined genotypic and phenotypic diversity among strains, which contain a striking number of variably detected chromosomal and plasmid genes that may be related to diverse clinical outcomes and adaptive changes favoring survival in different hosts (Pan et al., 2009; Huehn et al., 2010). However, direct correlations between genotypes and phenotypes would not be obvious, since indistinguishable genetic patterns have shown major differences in pathogenicity-associated phenotypes (Yim et al., 2010).

Because of the increasing impact of S. Enteritidis on public health in Chile and the unknown role of wildlife in its epidemiology, the aim of this work was to determine genotypic and phenotypic evidence of *S*. Enteritidis transmission among seabirds, poultry and humans in Chile.

## Materials and methods

### Isolates

Ninety *S*. Enteritidis isolates previously isolated from humans, poultry, and seabirds (*n* = 30 each) were analyzed (Table 1). Bacteria were grown routinely in liquid culture with Luria-Bertani (LB) medium (Bacto Tryptone, 10 g/L; Bacto Yeast Extract, 5 g/L; NaCl, 5 g/L) adjusted to pH 7 (NaHPO4/NaH2PO4 25 mM), at 37°C for 24 h, with shaking. When necessary, media were solidified by the addition of agar (15 g/L).

**Table 1:**
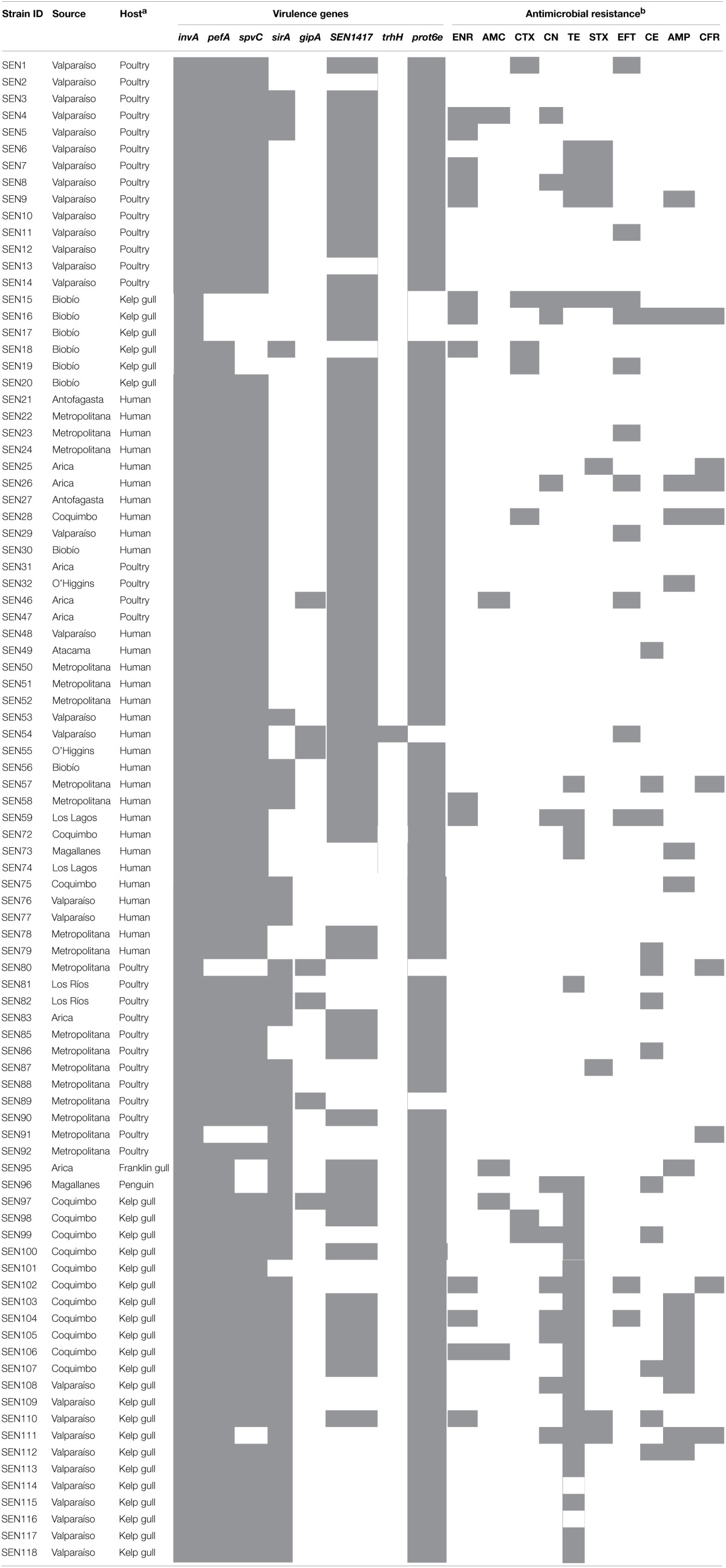
***Salmonella enterica* ser. Enteritidis strains utilized in this study**.

*Virulence genes and antimicrobial resistance phenotypes appear with shaded squares*.

*^a^Poultry, Gallus gallus; Human, Homo sapiens; Kelp gull, Larus dominicanus; Franklin' gull, Leucophaeus pipixcan; Penguin, Spheniscus magellanicus*.

*^b^AMC, Amoxicillin–clavulanic acid; AMP, Ampicillin; CTX, Cefotaxime; CFR, Cefadroxil; CE, Cefradine; EFT, Ceftiofur; ENR, Enrofloxacin; CN, Gentamicin; STX, Trimethoprim–sulfamethoxazol; TE, Tetracycline*.

### Genotypic assays

#### Virulotyping

This PCR based test was performed for the identification of genes *invA, pefA, spvC, sirA, gipA, SEN1417, trhH*, and *prot6e*, all of them associated with virulence that have been variably detected in *S*. Enteritidis (Pan et al., 2009; Huehn et al., 2010). After bacterial growth, PCR reactions were performed under standard conditions, as previously described (Fresno et al., 2013).

#### Pulse-field gel electrophoresis (PFGE)

This assay followed the PulseNet protocol (Ribot et al., 2006). The electrophoresis was performed using CHEF DRIII CHILER (Bio-Rad) equipment. DNA was digested with *Xba*I (50U/sample) endonuclease. PFGE patterns were analyzed with the GEL COMPAR II software (Applied Maths), using the Dice similarity coefficient with a 1% tolerance in band position.

#### Clustering

Results from virulotyping and PFGE were analyzed through the construction of a binary matrix using “1” for presence and “0” for absence of genes or bands from each isolate. Clusters were determined using via the unweighted pair group method (UPGMA) using TREECON software, and the discriminatory power (DP) was calculated with the Simpson's index of diversity, as reported in a previous study (Hunter and Gaston, 1988).

#### Multilocus sequence typing (MLST)

This procedure was based on sequencing seven housekeeping genes of *S. enterica* (Achtman et al., 2012), using the primers described in the MLST public database (http://mlst.warwick.ac.uk/mlst/). The sequence type was determined according to the scheme provided on this site.

### Phenotypic assays

#### Hydrogen peroxide and sodium nitrite survival assays

These assays were performed as described by Lu et al. (2002). Briefly, after an overnight growth of bacteria in LB broth the cultures were diluted 1/100 in LB pH 7 or LB pH 5 and challenged with 15 mM H_2_O_2_ or 10mM NaNO_2_, respectively. Then, cultures were incubated at 37°C with shaking for 30 min with H_2_O_2_, or for 3 h with NaNO_2_. For survival rate analysis, aliquots of culture were plated in triplicate before (time 0) and after challenge (30 min or 3 h). Survival was expressed as the percentage of colony forming units (CFU) after the assay; the count before the challenge was considered as 100%.

#### Acidic pH survival

After overnight growth in LB broth, bacteria were washed three times with LB pH 3 (citric acid 0.1 M), diluted 1/100 in the same medium and incubated with shaking at 37°C for 3 h. Survival analysis was performed as with H_2_O_2_ and NaNO_2_ assays, with CFU counts at 0 and 3 h.

#### Starvation survival assay

This procedure was based on published reports (Spector and Cubitt, 1992; O'neal et al., 1994), with some modifications. Briefly, bacterial isolates were inoculated into MOPS-buffered salts (MS) hiPCN (MS, 25 mM KH_2_PO_4_/K_2_HPO_4_ pH 7.4, 0.4% glucose, 15 mM NH_4_Cl) media, and then incubated for 16–18 h at 37°C. Cultures were washed with distilled water, diluted 1/10 in MS loPCN (MS, 1 mM KH_2_PO_4_/K_2_HPO_4_ pH 7.4, 0.2% glucose, 10 mM NH_4_Cl) media and incubated at 37°C up to OD_600_ 0.3–0.4. Then, 1 mL of this suspension was washed with distilled water, inoculated into 5 mL MS media and incubated at 37°C for 40 d. Aliquots of culture were plated in triplicate at different times. Survival was expressed as a percentage of CFU in relation with the maximal CFU count reached between day 0 and 5, which was considered 100%.

#### Survival within dendritic cells (DCs)

For this assay we used nine isolates, three from each host, which showed the highest resistance in the four previous survival assays. For selection, a survival ranking was performed in each assay, assigning 1 to the most susceptible and 90 to the most resistant strain. Then, an average ranking value was calculated for every isolate.

Human peripheral blood mononuclear-derived DCs were obtained from the buffy coats of six healthy donors and prepared as previously described (Vernal et al., 2008). For the infection, day 6 DCs were maintained in culture medium (RPMI-1640 with 10% fetal calf serum) and seeded into tissue culture plates at a concentration of 4 × 10^5^ cells per well. Exponential-phase (OD_600_, 0.6) grown bacteria were pelleted and suspended in the same medium. Aliquots of bacteria were added to DCs at a multiplicity of infection (MOI) of 50:1. After 1 h of infection, cells were washed three times with PBS, and incubated with cellular culture medium containing gentamicin (200 μg/mL). After additional incubation for 2 and 24 h, DCs were washed with PBS and permeabilized for 30 min with 0.1% Triton X-100, and the titers of intracellular bacteria were determined by serial dilution of cell lysates on LB agar plates. The percentage of survival was calculated at 2 h considering the initial inoculant as 100%, and at 24 h considering the CFU counted at 2 h as 100%.

### Ethics statement

The human DCs protocol included a written consent of all donors, which was approved by the University of Chile Clinical Hospital Scientific Ethics Committee (OAIC Reference #508/11, Exempt Resolution #570).

### Antimicrobial susceptibility

Antimicrobial susceptibility was evaluated by the disk diffusion method following CLSI criteria (CLSI, 2010). Antimicrobials tested were (μg/disk) ampicillin (10), amoxicillin–clavulanic acid (20/10), cefotaxime (30), gentamicin (10), trimethoprim–sulfamethoxazol (1.25/23.75), tetracycline (30), ciprofloxacin (5), cefradine (30), ceftiofur (30), and enrofloxacin (10) (Oxoid®). *Escherichia coli* ATCC 25922 was utilized as a control strain. The multidrug resistance condition was determined by the simultaneous resistance to three or more antimicrobial classes.

### Statistical analysis

Statistical analyses were performed using the ANOVA and Kruskal–Wallis test for independent samples. Categorical data and principal components analyses were performed using data from survival assays and hosts. These tests were calculated using INFOSTAT (2010v) software.

## Results

### Genotypic assays

PCR detection of virulence genes from 90 *S*. Enteritidis isolates showed 16 distinct virulotypes, resulting in a low discriminatory power (DP) methodology (0.773) that clustered 80% of isolates within three of these gene combinations (Table 2, Figure S1). Higher diversity was observed in isolates from poultry and seabirds (9 and 8 virulotypes, respectively) than in isolates detected in humans (5 virulotypes). In addition, virulotypes tended to associate with a specific host (*P* < 0.05), as the most frequent gene combination detected in poultry and human isolates is different from that identified in seabird isolates (virulotypes H and C, respectively. Table 2, Figure S1). This variation was also observed at genetic level, because *spvC* and *sirA* were differentially detected among hosts (*P* < 0.05), being the first most frequent in bacteria found in humans and poultry and the second in those isolated from seabirds (Table 3).

**Table 2:** **Virulence gene combinations (Virulotypes) of *Salmonella enterica* ser. Enteritidis strains and their frequency according to host**.

**Virulotypes**		**Host (N° of strains)**			
ID^*^	***invA-pefA-spvC-sirA-gipA-SEN1417-trhH-prot6e***	**Human (30)**	**Poultry (30)**	**Seabirds (30)**	**Total**
O	10000100	0	0	3	3
K	10010001	0	1	0	1
P	10011000	0	1	0	1
G	11000101	0	0	1	1
L	11010001	0	0	2	2
B	11010101	0	0	2	2
F	11100001	0	2	0	2
H	11100101	18	14	2	34
N	11101100	1	0	0	1
D	11101101	0	1	0	1
M	11101110	1	0	0	1
C	11110001	6	4	10	20
A	11110101	4	5	9	18
J	11111000	0	1	0	1
I	11111001	0	1	0	1
E	11111101	0	0	1	1

* *Letters were assigned correlatively according to the order in which isolates appear in the dendogram (Figure S1)*.

**Table 3:** **Frequency of virulence associated genes in *Salmonella enterica* ser. Enteritidis strains grouped by host**.

**Gene**	**Host^1^**			**Total *N* (%)**
	**Human *N* (%)**	**Poultry *N* (%)**	**Seabirds *N* (%)**	
*invA*	30 (100)	30 (100)	30 (100)	90 (100)
*pefA*	30 (100)	28 (93)	27 (90)	85 (94)
*spvC*	30 (100)^a^	28 (93)^a^	22 (73)^b^	80 (89)
*sirA*	10 (33)^a^	13 (43)^a^	24 (80)^b^	47 (52)
*gipA*	2 (7)	4 (13)	1 (3)	7 (8)
*SEN1417*	24 (80)	20 (67)	18 (60)	62 (69)
*trhH*	1 (3)	0 (0)	0 (0)	1 (1)
*prot6e*	28 (93)	28 (93)	27 (90)	83 (92)

1 *Different letters represents statistical differences between groups (p < 0.05)*.

**Figure 1:**
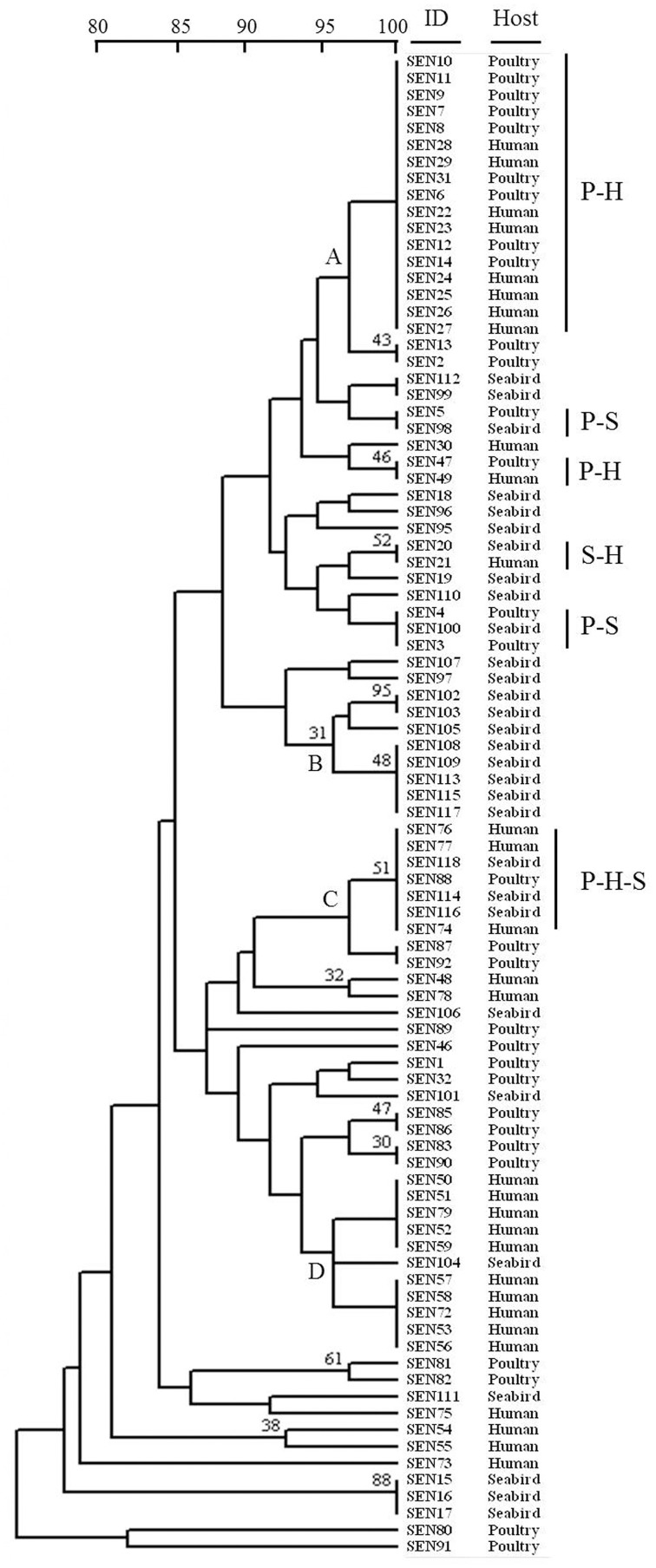
**Dendogram showing genetic similarities (%) between *Salmonella enterica* serovar Enteritidis strains resulting from combined PFGE and PCR data**. The ID and host are shown for each isolate. Clusters with at least five isolates sharing more than 95% similarity are indicated with letters A, B, C, and D. Mixed clusters are indicated with letters according to hosts (P, poultry; H, human; S, seabird). The tree was constructed using the UPGMA method with the software TREECON.

Through the PFGE procedure we obtained fingerprints with 8–12 bands, resulting in 10 major clusters (two or more isolates with identical PFGE profiles) that represent 89% of isolates (Figure S2). The DP of this method was 0.891, identifying five identical patterns between isolates from seabirds and humans. However, combined PFGE and PCR results showed the highest DP (0.949), with 16 major clusters that contain 70% of isolates. Six of these are mixed clusters, and two of them contain isolates from humans and seabirds (Figure 1). The MLST analyses determined that all *S*. Enteritidis isolates in this study belong to the sequence type (ST) 11.

### Phenotypic assays

**Figure 2:**
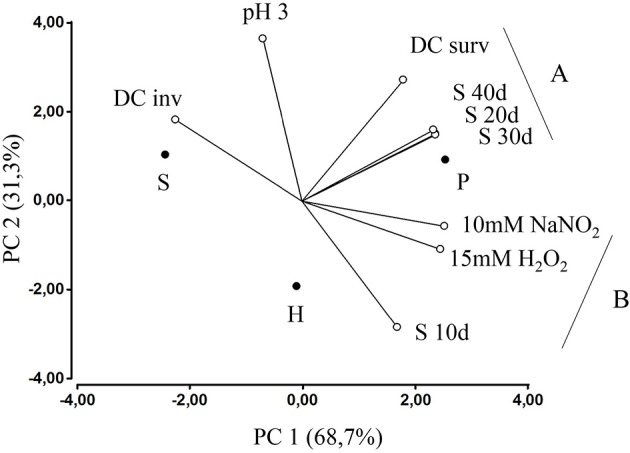
**Principal components analysis (PCA) showing the association between *Salmonella enterica* ser. Enteritidis hosts (displayed as filled circles) and results of phenotypic assays (displayed as linear axes and empty circles)**. In parenthesis appears the variability associated to each principal component (PC). S, seabirds; H, human; P, poultry; DC inv, invasion to dendritic cells; DC surv, survival within dendritic cells; pH3, survival to acidic pH; S, starvation assay (10–40 d, 10 to 40 days); 15 mM H_2_O_2_, survival to reactive oxygen species; 10 mM NaNO_2_, survival to reactive nitrogen species.

In pathogenicity-associated phenotypes there was a significant diversity (*P* < 0.05) of bacterial survival responses among isolates, which was observed in all these assays (data not shown). When results from individual strains were grouped and analyzed according to bacterial host, statistical differences (*P* < 0.05) were identified in starvation and dendritic cells survival assays (Table 4), in which isolates from poultry were the most resistant. Isolates from humans expressed higher resistance to short-term starvation (10 d), but later showed the steepest survival decrease and at 30 and 40 d showed similar CFU counts as bacteria belonging to seabirds. Among the top 10 most resistant isolates in each phenotypic assay, those recovered from poultry were consistently the most frequent (Figure S3). Within dendritic cells, isolates from humans were the most susceptible (Table 4). The relative survival performance of bacteria analyzed according to their hosts can be graphically seen in Figure 2. The poultry isolates have the closest position to most of assays, representing their highest survival capabilities in these challenges. Besides, some phenotypic variables are located forming two groups (A and B, Figure 2), depicting a high correlation between them.

**Table 4:** **Average survival percentages (%) of *Salmonella enterica* ser. Enteritidis strains in phenotypic assays grouped by host**.

**Assay^2^**	**Host^1^**			***P*-value**
	**Human**	**Poultry**	**Seabirds**	
pH3	109.43	113.98	116.08	0.70
H_2_O_2_	19.07	21.09	13.70	0.71
NaNO_2_	24.29	27.30	19.80	0.85
Starvation 10 d	30.64^a^	23.39^a^	6.19^b^	0.0004
Starvation 20 d	2.31^a^	16.29^a^	0.91^b^	0.0005
Starvation 30 d	0.66^a^	2.73^b^	0.43^a^	0.0022
Starvation 40 d	0.39^a^	1.69^b^	0.31^a^	0.029
DCs invasion	1.4E-05	1.2E-05	4.2E-05	0.66
DCs survival	1.59^a^	27.17^c^	9.49^b^	0.0036

1 *Different letters represents statistical differences between groups*.

2 *pH3, H_2_O_2_, NaNO_2_ and starvation assays: 30 isolates from every host. Assays with dendritic cells: 3 isolates from every host. Each assay was performed in three independent experiments*.

**Figure 3:**
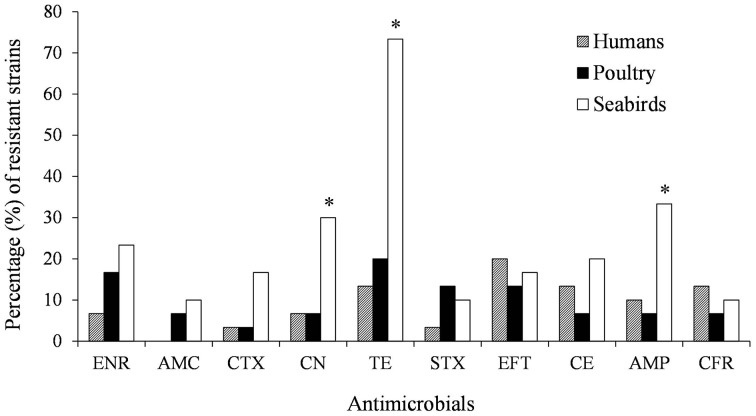
Percentages (%) of antimicrobial resistant *Salmonella enterica* ser. Enteritidis strains grouped according to their hosts. (^*^P < 0.05). AMC, Amoxicillin–clavulanic acid; AMP, Ampicillin; CTX, Cefotaxime; CFR, Cefadroxil; CE, Cefradine; EFT, Ceftiofur; ENR, Enrofloxacin; CN, Gentamicin; STX, Trimethoprim–sulfamethoxazol; TE, Tetracycline.

Resistance to gentamycin, tetracycline and ampicillin was statistically associated with *S*. Enteritidis isolates from seabirds (*P* < 0.05, Figure 3). Additionally, we detected multidrug resistance (simultaneous resistance to three or more different CLSI antimicrobial clasess) in 13 (43%) isolates recovered from seabirds; in contrast, only 3 (10%) and 4 (13%) human and poultry isolates showed multidrug resistance, respectively (Table 1).

Significant associations (*P* < 0.05) between genotypic and phenotypic results were detected when isolates from major clusters (A–D in Figure 1) were compared (Table 5). The cluster A, which only contains isolates from humans and poultry, shows the lowest survival to acidic pH. The cluster C, which is composed by isolates from the three hosts, shows the lowest survival to nitrite-derived free radicals and the highest survival to short-term starvation (Table 5).

**Table 5:** **Survival percentages (%) of *Salmonella enterica* ser. Enteritidis genotypic clusters**.

**Assay**	**Genotypic cluster^1^**				***P*-value**
	***A***	***B***	***C***	***D***	
pH3	76.4^a^	133.6^b^	157.8^b^	132.6^b^	0.0002
H_2_O_2_	18.8	12.1	11.5	26.3	0.1989
NaNO_2_	33.9^a^	20.5^ab^	5.5^b^	34.7^a^	0.0094
Starvation 10 d	6.7^a^	6.5^a^	61.4^b^	17.4^a^	<0.0001
Starvation 20 d	2.4	1.0	17.8	1.9	0.1436
Starvation 30 d	2.4	0.5	2.0	0.6	0.1462
Starvation 40 d	1.6	0.2	1.5	0.3	0.2012

1 *Isolates constituting clusters appear in Figure 1. Different letters represents statistical differences between clusters*.

## Discussion

Wild birds have received attention from sanitary authorities because of both their association with several highly transmissible zoonotic pathogens and their ability to disseminate agents with a wide host range, over wide geographical areas (Hubalek, 2004).

In this work, *S*. Enteritidis isolates from different hosts have been characterized and compared, attempting to elucidate the human-animal interface of this bacterium in Chile. The existing evidence has established a major transmission chain of *S*. Enteritidis between poultry and humans through food consumption (Fernandez et al., 2003; Fica et al., 2012). However, increasing reports of human cases cannot be solely explained by this link, since prevention efforts have been progressively incorporated in recent years. The use of genotypic and phenotypic methods for bacterial typing was our experimental strategy to shed light on the role of seabirs as a third factor in *Salmonella* epidemiology.

Virulotyping rapidly allows the discrimination of isolates with diverse pathogenic potential or host specificities (Huehn et al., 2010). Despite the low DP of the procedure (0.773), we identified 16 gene combinations heterogeneously distributed among hosts (Table 2, Figure S1). Variations in the gene repertoire were mainly associated with *spvC* and *sirA* sequences (*P* < 0.05) (Table 3). SpvC is a Type III secretion system (T3SS) effector involved in immune evasion and dissemination (Haneda et al., 2012), which importance for the colonization of humans seems represented by our 100% *spvC* detection in isolates from this host (Table 3). The *spvC* instability is due to its location on the pSLT virulence plasmid (Fabrega and Vila, 2013), which characterizes the serotypes Enteritidis and Typhimurium. However, the other analyzed pSLT encoded sequences, *pefA* and *prot6e*, were not statistically different among hosts, suggesting that the plasmid gain or loss is not a unique variability mechanism for these sequences. On the other hand, the *sirA* gene encodes a global regulator of virulence, motility and biofilm formation (Teplitski et al., 2006). Its significant association (*P* < 0.05) with bacteria isolated from seabirds suggest particular requirements for *S*. Enteritidis attachment, survival and transmission in marine environments.

Because of its high DP and reproducibility for bacterial typing, the PFGE technique has been utilized to compare *Salmonella* strains isolated from a diversity of hosts and substrates (Ribot et al., 2006; Zheng et al., 2011; Sandt et al., 2013). In this work, the combination of *Xba*I PFGE with virulotyping has allowed the best DP (0.949), comparable to PFGE using a combination of *Xba*I and *Bln*I restriction enzymes (Zou et al., 2012). This procedure is showing several indistinguishable patterns among human, poultry and seabirds isolates (Figure 1). Moreover, the MLST analysis has classified all analyzed isolates as belonging to ST11, which is distributed worldwide (http://mlst.warwick.ac.uk/mlst/) and has also been associated with prevalent phage types (Pan et al., 2009),

Overall, genotypic data suggests that wild birds are sharing bacteria, whether directly or indirectly, with poultry and humans, participating in the transmission cycle of *S*. Enteritidis in Chile.

Phenotypic assays were performed in order to determine the pathogenic potential of *Salmonella* isolates from different hosts. The analyzed conditions are mainly found when bacteria face the gastrointestinal lumen upon entering a host via ingestion and within the phagolysosomal environment in phagocytic cells (Behnsen et al., 2015), although survival as free-living bacteria in water, soil or within protozoa in extra-host settings represents a similar challenge (Spector and Kenyon, 2012).

In this work we were able to determine differences among *S*. Enteritidis strains (*P* < 0.05) in all phenotypic assays. When results were grouped according to host source, starvation survival constitutes the unique *in vitro* assay that demonstrates differences (*P* < 0.05) among hosts (Table 4), showing that seabird isolates are the most susceptible to nutrient deprivation at 37°C. The human isolates demonstrated a critical downshift between 10 and 30 days (Table 4), suggesting a better adaptation to shorter rather than longer periods of starvation. Moreover, poultry strains not only had the highest survival rate to starvation during the entire experiment, but also showed the highest resistance when all *in vitro* assays were considered. Because of that, they are the best represented among the top 10 most resistant strains within every survival assay (Figure S3).

Unexpectedly, genotypic clustering at 95% similarity (Figure 1) was associated with three survival phenotypes (Table 5) in contrast to previous reports with this serotype which, although not using the same methods, did not find such association (Betancor et al., 2009; Yim et al., 2010). The cluster A appear the most defective in pH 3 assay, probably due to absence of seabirds' isolates, which showed the best (non-statistical, *P* > 0.05) fitness in this challenge (Figure 2, Table 4). Interestingly, the cluster C, which is the unique cluster composed by isolates from the three hosts, is associated with the lowest performance in NaNO_2_-derived free radicals and the highest survival to a short-term starvation condition (Table 5). Whether the transmission between aquatic and terrestrial hosts, suggested by the high genetic similitude of bacteria within cluster C, could be facilitated by such combination of phenotypic responses, is a question that remains to be elucidated.

During the infective process it has been determined that a critical survival challenge facing *S*. Enteritidis inside its host is DCs, because it cannot replicate within these cells as well as in macrophages after invasion (Swart and Hensel, 2012). In order to determine a correlation between *in vitro* phenotypes and survival within human dendritic cells, we performed a gentamicin protection assay using differentiated human peripheral blood monocytes infected with the most resistant *Salmonella* strains isolated from the three hosts. Interestingly, the highest correlation was found with starvation assays at 20 (*r* = 0.93), 30 (*r* = 0.92), and 40 days (*r* = 0.94), suggesting this *in vitro* assay is a predictor of bacterial behavior in the intracellular environment and the pathogenic potential in the human host. In this assay, poultry isolates have again shown the highest survival rate, and unexpectedly, human isolates were the most susceptible (Table 4). Consistently, poultry strains have shown the highest survival rates in most assays, suggesting an increased virulence. It is probable that the high contact rates within poultry flocks promote selection of the most rapidly replicating and most virulent clones, because the transmission to other animals will occur no matter the clinical outcome in infected hosts (Berngruber et al., 2013). When represented graphically (Figure 2), the relationship between survival to pathogenicity-associated stresses and the source of *Salmonella* isolates highlights the mentioned differential bacterial performances. Furthermore, two groups of assays are formed according to their correlations, one including the short-term starvation survival (S 10 d) with resistance against free radicals (B, Figure 2), and the other including long-term starvation survival (S 20, 30, 40 d) with survival within DCs (A, Figure 2). This reflects that within each group, the bacterial mechanisms to resist these challenges are sharing stimuli, regulatory factors or effectors, in agreement with previous studies in other bacteria (Watson et al., 1998; Cuny et al., 2005).

The high amount of prescribed antimicrobials in human and veterinary medicine represent a global concern because of the spread of antimicrobial resistant infectious pathogens (WHO, 2014). In general, *S*. Enteritidis isolates have shown low resistance levels against antimicrobials (Huehn et al., 2010; Sandt et al., 2013), contrasting with our results that show host-associated variability in this matter and suggest an overall high frequency of drug resistance and MDR phenotypes, especially in wild birds. From antimicrobials tested, the highest frequencies of resistances were detected against ceftiofur in humans (20%) and against tetracycline in poultry (20%) and wild birds (73%). This constitutes a concerning situation since ceftiofur is prescribed for animal use only, suggesting transmission of resistant strains from animals to humans. Isolates belonging to seabirds expressed significantly higher percentages of resistance (*P* < 0.05) than human and poultry isolates with antimicrobials gentamicin (30%), ampicillin (33%), and tetracycline (73%) (Figure 3). The long established environmental persistence of tetracyclines (Hamscher et al., 2002), could explain the high resistance to this antimicrobial. The environment can persistently spread resistant bacteria and sublethal antimicrobial concentrations that, derived from anthropogenic activities (mainly animal farms and wastewater), can select for resistance (Tello et al., 2012; Andersson and Hughes, 2014). Besides, the appearing of virulence-resistance plasmids that encode antimicrobial resistance and virulence factors, determines co-selection of these functions even in the absence of antimicrobials (Rodriguez et al., 2012b; Gullberg et al., 2014), which could also explain our findings. In any case, these situations represents a potential risk to both public and animal health (Wellington et al., 2013), and justifies the study of these hosts as bio-indicators of resistance traits dispersion into the environment (da Costa et al., 2013).

Altogether, genotypic and phenotypic evidence gathered in this study suggest that *S*. Enteritidis is circulating among wildlife, domestic animals and humans, with human beings participating as incidental (spill-over) hosts. Seabirds can be reservoirs of *Salmonella* strains with potential risk to public and animal health, and could partially explain the progressive rise in the incidence of these serotype-associated outbreaks. Whether such transmission among hosts is direct or indirect is a question that should be addressed in future analyses. Our results support the establishment of biosecurity measures for animal farms and systematic *Salmonella* surveillance campaigns in seabirds, determining not only the genetic similarities of bacterial strains but also their pathogenic potential in susceptible hosts.

### Conflict of interest statement

The authors declare that the research was conducted in the absence of any commercial or financial relationships that could be construed as a potential conflict of interest.

## Supplementary material

Figure S1 **Dendogram showing genetic similarities (%) between Salmonella enterica ser. Enteritidis strains resulting from PCR data**. For each strain, detected genes (black boxes) and host are also shown. The tree was constructed using the UPGMA method with the software TREECON (1000 replicates).

Figure S2 **Dendogram showing genetic similarities (%) between Salmonella enterica ser. Enteritidis strains resulting from PFGE XbaI data**. For each strain, the genotypic pattern and host are also shown. The tree was constructed using the UPGMA method with the GEL COMPAR II software (1000 replicates) with a 1% of tolerance in band position.

Figure S3 **Number of Salmonella enterica ser. Enteritidis strains within the top 10 most resistant isolates in every stressful challenge, according to their host source**.

## References

[B1] Achtman M. Wain J. Weill F. X. Nair S. Zhou Z. Sangal V.. (2012). Multilocus sequence typing as a replacement for serotyping in *Salmonella enterica*. PLoS Pathog. 8:e1002776. 10.1371/journal.ppat.1002776 22737074 PMC3380943

[B2] Andersson D. I. Hughes D. (2014). Microbiological effects of sublethal levels of antibiotics. Nat. Rev. Microbiol. 12, 465–478. 10.1038/nrmicro3270 24861036

[B3] Behnsen J. Perez-Lopez A. Nuccio S. P. Raffatellu M. (2015). Exploiting host immunity: the Salmonella paradigm. Trends Immunol. 36, 112–120. 10.1016/j.it.2014.12.003 25582038 PMC4323876

[B4] Berngruber T. W. Froissart R. Choisy M. Gandon S. (2013). Evolution of virulence in emerging epidemics. PLoS Pathog. 9:e1003209. 10.1371/journal.ppat.1003209 23516359 PMC3597519

[B5] Betancor L. Yim L. Fookes M. Martinez A. Thomson N. R. Ivens A.. (2009). Genomic and phenotypic variation in epidemic-spanning *Salmonella enterica* serovar Enteritidis isolates. BMC Microbiol. 9:237. 10.1186/1471-2180-9-237 19922635 PMC2784474

[B6] CLSI. (2010). Performance Standards for Antimicrobial Susceptibility Testing; Twentieth Informational Supplement. Wayne, PA: Clinical and Laboratory Standards Institute.

[B7] Cuny C. Dukan L. Fraysse L. Ballesteros M. Dukan S. (2005). Investigation of the first events leading to loss of culturability during *Escherichia coli* starvation: future nonculturable bacteria form a subpopulation. J. Bacteriol. 187, 2244–2248. 10.1128/JB.187.7.2244-2248.2005 15774865 PMC1065215

[B8] da Costa P. M. Loureiro L. Matos A. J. (2013). Transfer of multidrug-resistant bacteria between intermingled ecological niches: the interface between humans, animals and the environment. Int. J. Environ. Res. Public Health 10, 278–294. 10.3390/ijerph10010278 23343983 PMC3564142

[B9] Dhama K. Mahendran M. Tomar S. (2008). Pathogens transmitted by migratory birds: threat perceptions to poultry health and production. Int. J. Poultry Sci. 7, 516–525 10.3923/ijps.2008.516.525

[B10] Fabrega A. Vila J. (2013). *Salmonella enterica* serovar Typhimurium skills to succeed in the host: virulence and regulation. Clin. Microbiol. Rev. 26, 308–341. 10.1128/CMR.00066-12 23554419 PMC3623383

[B11] Fernandez J. Fica A. Ebensperger G. Calfullan H. Prat S. Fernandez A.. (2003). Analysis of molecular epidemiology of chilean *Salmonella enterica* serotype enteritidis isolates by pulsed-field gel electrophoresis and bacteriophage typing. J. Clin. Microbiol. 41, 1617–1622. 10.1128/JCM.41.4.1617-1622.2003 12682153 PMC153903

[B12] Fica A. Acosta G. Dabanch J. Perret C. Torres M. Lopez J.. (2012). [Salmonellosis outbreaks and the size and role of the Chilean State]. Rev. Chilena Infectol. 29, 207–214. 10.4067/S0716-10182012000200014 22689037

[B13] Fresno M. Barrera V. Gornall V. Lillo P. Paredes N. Abalos P.. (2013). Identification of diverse Salmonella Serotypes, Virulotypes, and antimicrobial resistance phenotypes in waterfowl from chile. Vector Borne Zoonotic Dis. 13, 884–887. 10.1089/vbz.2013.1408 24107205 PMC3868272

[B14] Gruszynski K. Pao S. Kim C. Toney D. M. Wright K. Colon A.. (2014). Evaluating gulls as potential vehicles of *Salmonella enterica* serotype Newport (JJPX01.0061) contamination of tomatoes grown on the eastern shore of Virginia. Appl. Environ. Microbiol. 80, 235–238. 10.1128/AEM.02809-13 24141129 PMC3911029

[B15] Gullberg E. Albrecht L. M. Karlsson C. Sandegren L. Andersson D. I. (2014). Selection of a multidrug resistance plasmid by sublethal levels of antibiotics and heavy metals. MBio 5, e01918–e01914. 10.1128/mBio.01918-14 25293762 PMC4196238

[B16] Hamscher G. Sczesny S. Hoper H. Nau H. (2002). Determination of persistent tetracycline residues in soil fertilized with liquid manure by high-performance liquid chromatography with electrospray ionization tandem mass spectrometry. Anal. Chem. 74, 1509–1518. 10.1021/ac015588m 12033238

[B17] Haneda T. Ishii Y. Shimizu H. Ohshima K. Iida N. Danbara H.. (2012). Salmonella type III effector SpvC, a phosphothreonine lyase, contributes to reduction in inflammatory response during intestinal phase of infection. Cell. Microbiol. 14, 485–499. 10.1111/j.1462-5822.2011.01733.x 22188134

[B18] Hendriksen R. S. Vieira A. R. Karlsmose S. Lo Fo Wong D. M. Jensen A. B. Wegener H. C.. (2011). Global monitoring of Salmonella serovar distribution from the World Health Organization Global Foodborne Infections Network Country Data Bank: results of quality assured laboratories from 2001 to 2007. Foodborne Pathog. Dis. 8, 887–900. 10.1089/fpd.2010.0787 21492021

[B19] Horton R. A. Wu G. Speed K. Kidd S. Davies R. Coldham N. G.. (2013). Wild birds carry similar *Salmonella enterica* serovar Typhimurium strains to those found in domestic animals and livestock. Res. Vet. Sci. 95, 45–48. 10.1016/j.rvsc.2013.02.008 23481141

[B20] Hubalek Z. (2004). An annotated checklist of pathogenic microorganisms associated with migratory birds. J. Wildl Dis. 40, 639–659. 10.7589/0090-3558-40.4.639 15650082

[B21] Huehn S. La Ragione R. M. Anjum M. Saunders M. Woodward M. J. Bunge C.. (2010). Virulotyping and antimicrobial resistance typing of *Salmonella enterica* serovars relevant to human health in Europe. Foodborne Pathog. Dis. 7, 523–535. 10.1089/fpd.2009.0447 20039795

[B22] Hunter P. R. Gaston M. A. (1988). Numerical index of the discriminatory ability of typing systems: an application of Simpson's index of diversity. J. Clin. Microbiol. 26, 2465–2466. 3069867 10.1128/jcm.26.11.2465-2466.1988 PMC266921

[B23] Jackson B. R. Griffin P. M. Cole D. Walsh K. A. Chai S. J. (2013). Outbreak-associated *Salmonella enterica* serotypes and food Commodities, United States, 1998-2008. Emerg. Infect. Dis. 19, 1239–1244. 10.3201/eid1908.121511 23876503 PMC3739514

[B24] Lopez-Martin J. Junod T. Riquelme F. Contreras C. Gonzalez-Acuna D. (2011). [Detection of Salmonella and Mycobacterium species in seagulls captured in Talcahuano, Chile]. Rev. Med. Chil. 139, 1496–1502. 10.4067/S0034-98872011001100017 22446658

[B25] Lu S. Killoran P. B. Fang F. C. Riley L. W. (2002). The global regulator ArcA controls resistance to reactive nitrogen and oxygen intermediates in *Salmonella enterica* serovar Enteritidis. Infect. Immun. 70, 451–461. 10.1128/IAI.70.2.451-461.2002 11796570 PMC127680

[B26] O'neal C. R. Gabriel W. M. Turk A. K. Libby S. J. Fang F. C. Spector M. P. (1994). RpoS is necessary for both the positive and negative regulation of starvation survival genes during phosphate, carbon, and nitrogen starvation in *Salmonella typhimurium*. J. Bacteriol. 176, 4610–4616. 8045891 10.1128/jb.176.15.4610-4616.1994 PMC196281

[B27] Pan Z. Carter B. Nuñez-García J. Abuoun M. Fookes M. Ivens A.. (2009). Identification of genetic and phenotypic differences associated with prevalent and non-prevalent Salmonella Enteritidis phage types: analysis of variation in amino acid transport. Microbiology 155, 3200–3213. 10.1099/mic.0.029405-0 19574306

[B28] Pennycott T. W. Park A. Mather H. A. (2006). Isolation of different serovars of *Salmonella enterica* from wild birds in Great Britain between 1995 and 2003. Vet. Rec. 158, 817–820. 10.1136/vr.158.24.817 16782854

[B29] Reche M. P. Jimenez P. A. Alvarez F. Garcia De Los Rios J. E. Rojas A. M. De Pedro P. (2003). Incidence of salmonellae in captive and wild free-living raptorial birds in central Spain. J. Vet. Med. B Infect. Dis. Vet. Public Health 50, 42–44. 10.1046/j.1439-0450.2003.00623.x 12710500

[B30] Ribot E. M. Fair M. A. Gautom R. Cameron D. N. Hunter S. B. Swaminathan B.. (2006). Standardization of pulsed-field gel electrophoresis protocols for the subtyping of *Escherichia coli* O157:H7, Salmonella, and Shigella for PulseNet. Foodborne Pathog Dis 3, 59–67. 10.1089/fpd.2006.3.59 16602980

[B31] Rodriguez F. Moreno J. Ortega R. Mathieu C. Garcia A. Cerda-Leal F.. (2012a). Evidence for kelp gulls (*Larus dominicanus*) and Franklin's Gulls (*Leucophaeus pipixcan*) as carriers of salmonella by real-time polymerase chain reaction. J. Wildl. Dis. 48, 1105–1108. 10.7589/2012-04-104 23060519

[B32] Rodriguez I. Rodicio M. R. Guerra B. Hopkins K. L. (2012b). Potential international spread of multidrug-resistant invasive *Salmonella enterica* serovar enteritidis. Emerg. Infect. Dis. 18, 1173–1176. 10.3201/eid1807.120063 22709653 PMC3376808

[B33] Sandt C. H. Fedorka-Cray P. J. Tewari D. Ostroff S. Joyce K. M'ikanatha N., M. (2013). A comparison of non-typhoidal Salmonella from humans and food animals using pulsed-field gel electrophoresis and antimicrobial susceptibility patterns. PLoS ONE 8:e77836. 10.1371/journal.pone.0077836 24204990 PMC3813714

[B34] Skov M. N. Madsen J. J. Rahbek C. Lodal J. Jespersen J. B. Jorgensen J. C.. (2008). Transmission of Salmonella between wildlife and meat-production animals in Denmark. J. Appl. Microbiol. 105, 1558–1568. 10.1111/j.1365-2672.2008.03914.x 19146492

[B35] Spector M. P. Cubitt C. L. (1992). Starvation-inducible loci of *Salmonella typhimurium*: regulation and roles in starvation-survival. Mol. Microbiol. 6, 1467–1476. 10.1111/j.1365-2958.1992.tb00867.x 1320726

[B36] Spector M. P. Kenyon W. J. (2012). Resistance and survival strategies of *Salmonella enterica* to environmental stresses. Food Res. Int. 45, 455–481 10.1016/j.foodres.2011.06.056

[B37] Sturm N. Abalos P. Fernandez A. Rodriguez G. Oviedo P. Arroyo V.. (2011). *Salmonella enterica* in pinnipeds, Chile. Emerg. Infect. Dis. 17, 2377–2378. 10.3201/eid1712.111103 22172111 PMC3311217

[B38] Swart A. L. Hensel M. (2012). Interactions of *Salmonella enterica* with dendritic cells. Virulence 3, 660–667. 10.4161/viru.22761 23221476 PMC3545948

[B39] Tello A. Austin B. Telfer T. C. (2012). Selective pressure of antibiotic pollution on bacteria of importance to public health. Environ. Health Perspect. 120, 1100–1106. 10.1289/ehp.1104650 22571927 PMC3440082

[B40] Teplitski M. Al-Agely A. Ahmer B. M. (2006). Contribution of the SirA regulon to biofilm formation in *Salmonella enterica* serovar Typhimurium. Microbiology 152, 3411–3424. 10.1099/mic.0.29118-0 17074910

[B41] Vernal R. Leon R. Herrera D. Garcia-Sanz J. A. Silva Sanz M. (2008). Variability in the response of human dendritic cells stimulated with Porphyromonas gingivalis or Aggregatibacter actinomycetemcomitans. J. Periodontal. Res. 43, 689–697. 10.1111/j.1600-0765.2007.01073.x 19031495

[B42] Watson S. P. Clements M. O. Foster S. J. (1998). Characterization of the starvation-survival response of Staphylococcus aureus. J. Bacteriol. 180, 1750–1758. 9537371 10.1128/jb.180.7.1750-1758.1998 PMC107086

[B43] Wellington E. M. Boxall A. B. Cross P. Feil E. J. Gaze W. H. Hawkey P. M.. (2013). The role of the natural environment in the emergence of antibiotic resistance in gram-negative bacteria. Lancet Infect. Dis. 13, 155–165. 10.1016/S1473-3099(12)70317-1 23347633

[B44] WHO. (2014). Antimicrobial Resistance: Global Report on Surveillance. [Online]. World Health Organization. Available online at: http://apps.who.int/iris/bitstream/10665/112642/1/9789241564748_eng.pdf (Accessed May 2014).

[B45] Yim L. Betancor L. Martinez A. Giossa G. Bryant C. Maskell D. (2010). Differential phenotypic diversity among epidemic-spanning *Salmonella enterica* serovar enteritidis isolates from humans or animals. Appl. Environ. Microbiol. 76, 6812–6820 10.1128/AEM.00497-10 20802078 PMC2953042

[B46] Zheng J. Keys C. E. Zhao S. Ahmed R. Meng J. Brown E. W. (2011). Simultaneous analysis of multiple enzymes increases accuracy of pulsed-field gel electrophoresis in assigning genetic relationships among homogeneous Salmonella strains. J. Clin. Microbiol. 49, 85–94. 10.1128/JCM.00120-10 20980570 PMC3020462

[B47] Zou M. Keelara S. Thakur S. (2012). Molecular characterization of *Salmonella enterica* serotype Enteritidis isolates from humans by antimicrobial resistance, virulence genes, and pulsed-field gel electrophoresis. Foodborne Pathog. Dis. 9, 232–238. 10.1089/fpd.2011.1012 22283616

