# Supplementary figures and images for: Genetic and phenotypic evidence of the Salmonella enterica serotype Enteritidis human-animal interface in Chile
Source: Front Microbiol. 2015 May 15;6:464. doi: 10.3389/fmicb.2015.00464 (PMC4432690; doi:10.3389/fmicb.2015.00464)

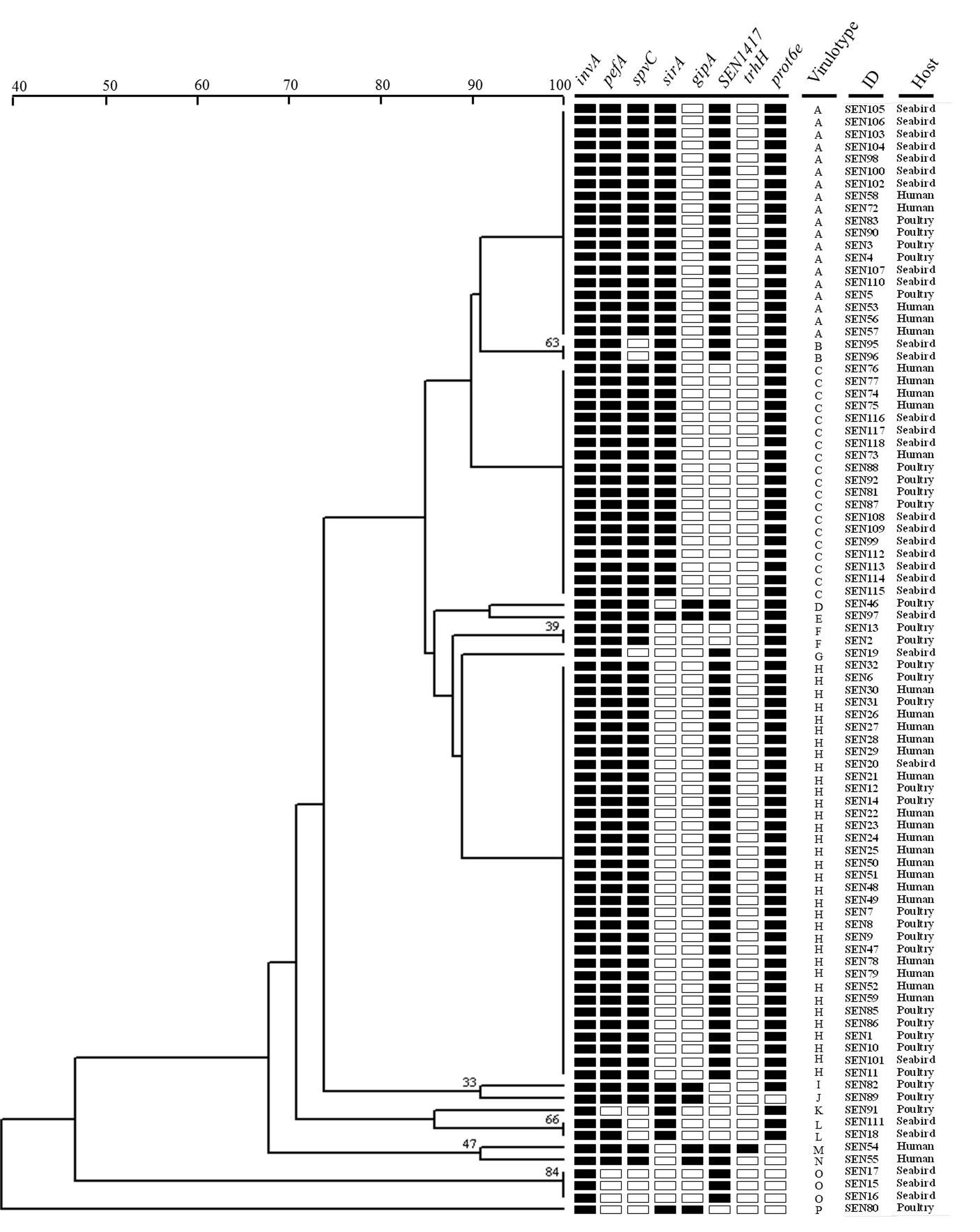

Supplement: Figure S1 — Dendogram showing genetic similarities (%) between Salmonella enterica ser. Enteritidis strains resulting from PCR data. For each strain, detected genes (black boxes) and host are also shown. The tree was constructed using the UPGMA method with the software TREECON (1000 replicates). [file Image1.JPEG]

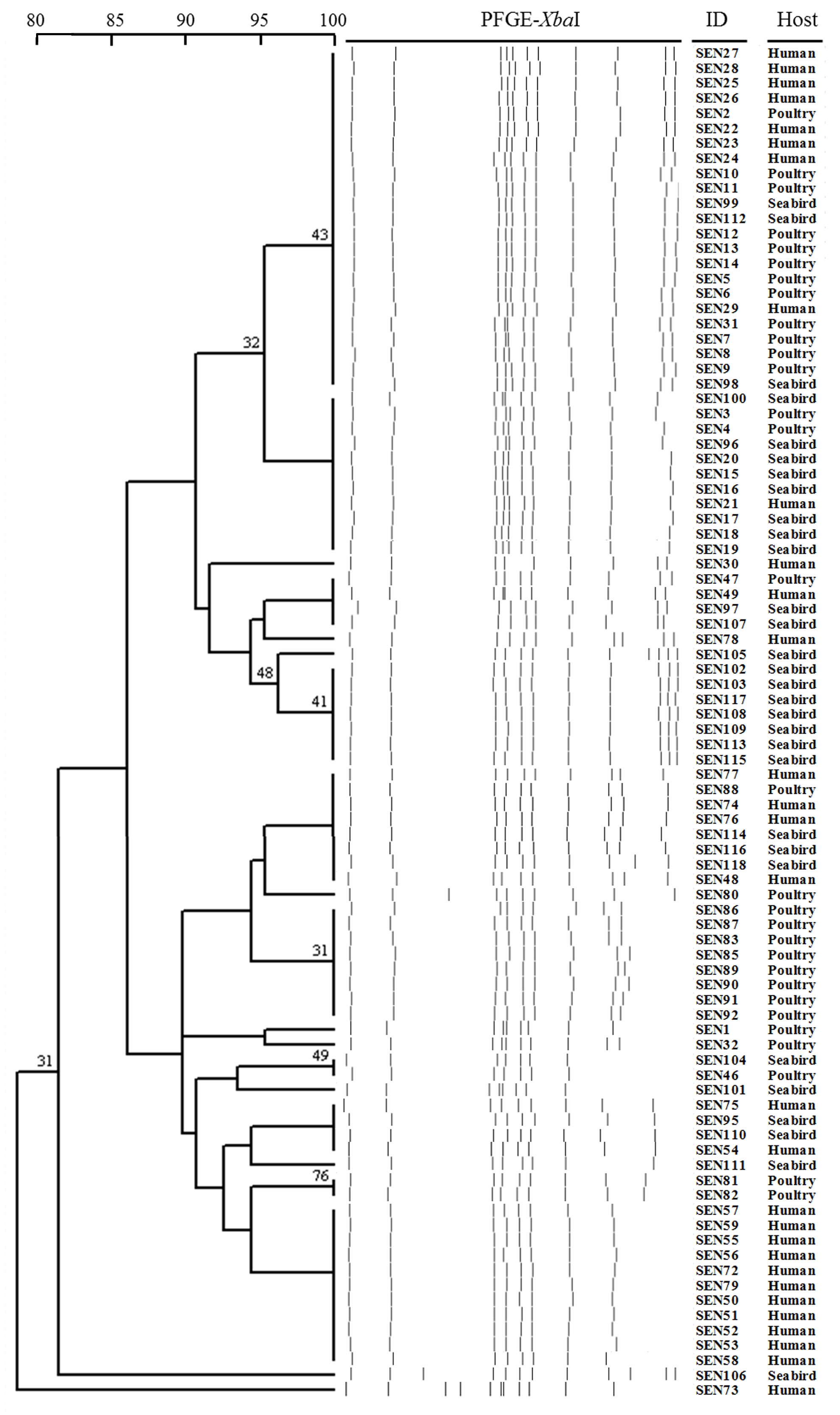

Supplement: Figure S2 — Dendogram showing genetic similarities (%) between Salmonella enterica ser. Enteritidis strains resulting from PFGE XbaI data. For each strain, the genotypic pattern and host are also shown. The tree was constructed using the UPGMA method with the GEL COMPAR II software (1000 replicates) with a 1% of tolerance in band position. [file Image2.JPEG]

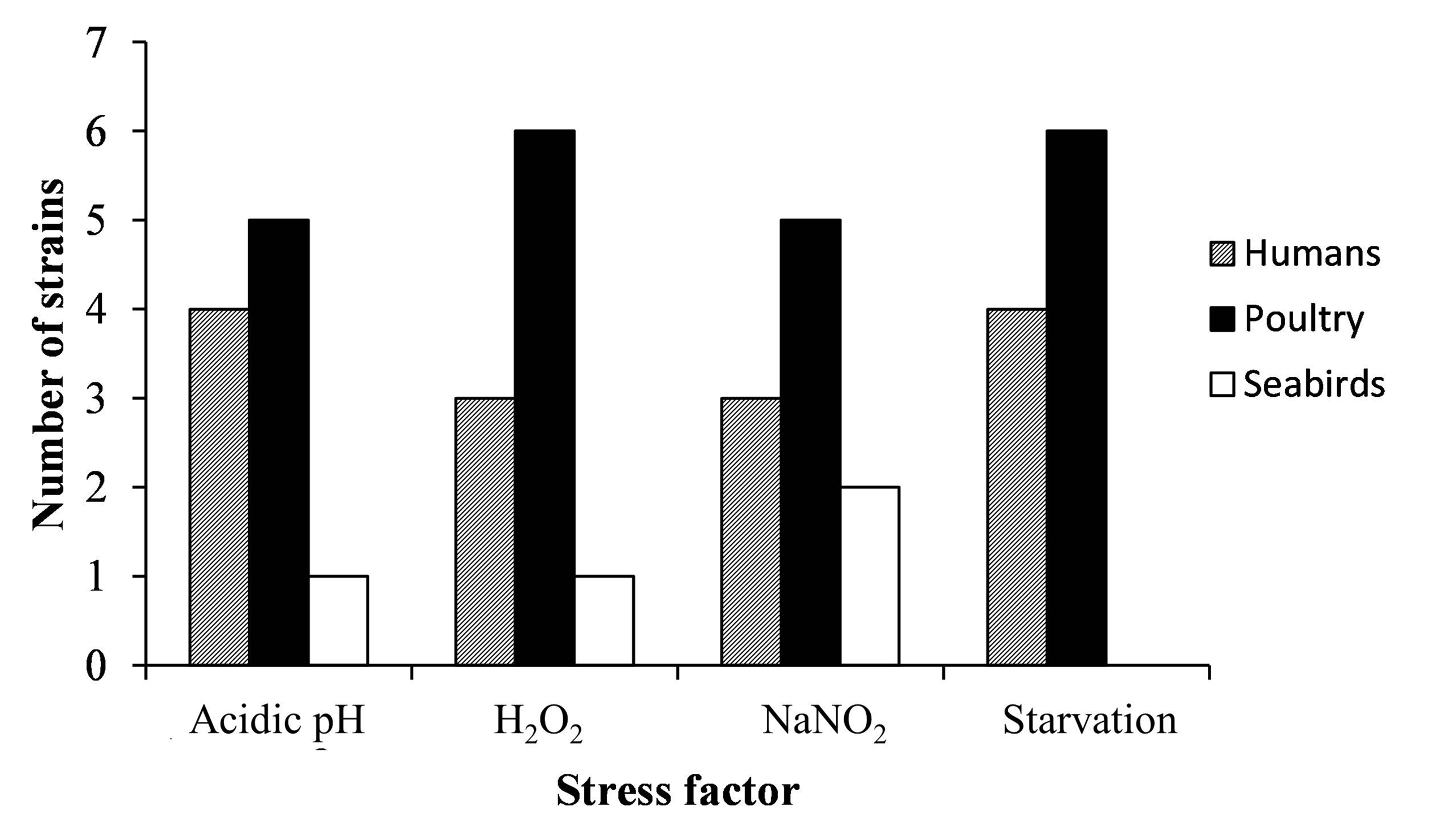

Supplement: Figure S3 — Number of Salmonella enterica ser. Enteritidis strains within the top 10 most resistant isolates in every stressful challenge, according to their host source. [file Image3.JPEG]
